# Sensitivity and specificity of high frequency ultrasound score (DCEC) in diabetic peripheral neuropathy

**DOI:** 10.1007/s40200-022-01080-6

**Published:** 2022-08-05

**Authors:** Hailun Huang, Chao Tang, Mi Li, Jing Huang, Yan Li, Shan Wu

**Affiliations:** 1 Department of Neurology, Affiliated Hospital of Guizhou Medical University, 550001 Guiyang, China; 2 Department of Neuropathy, Affiliated Hospital of Guizhou Medical University, No. 28 of Guiyi Street, Yunyan District, 550004 Guiyang, China

## Abstract

**Objectives:**

To summarize the ultrasonic characteristics of peripheral nerve damage in type 2 diabetes and to verify the diagnostic value of DCEC score for DPN.

**Methods:**

A total of 289 patients with type 2 diabetes evaluated peripheral neuropathy with neuroultrasound and nerve conduction at the Affiliated Hospital of Guizhou Medical University from June 2016 to June 2020. According to the diagnostic criteria from 2017 guidelines of China, 289 patients with type 2 diabetes were divided into three groups: DPN group: 203 cases; subclinical group: 48 cases; and non-DPN group: 38 cases. Kruskal Wallis test was used to identify the differences and characteristics of ultrasound scores between the all groups. The best cut-off value, sensitivity and specificity of DCEC score were obtained by receiver operator characteristic curve. Taking the diagnostic standard of diabetes peripheral neuropathy as the “gold standard”, the best diagnostic threshold, sensitivity and specificity were obtained by drawing the ROC curve of DCEC score, and then the diagnostic value of DCEC score for DPN was verified

**Results:**

Compared with non-DPN group, DCEC score in DPN group was significantly higher (P < 0.05). Otherwise,according to the ROC curve, the best cut-off value of DCEC score for DPN diagnosis was 12.5 (sensitivity 69.7%, specificity 71.1%).

**Conclusions:**

The DCEC score system can effectively diagnose DPN with length-dependence,mainly including the increase of definition score.

- **Abbreviations**: DPN: Diabetic Peripheral Neuropathy; DCEC: Definition, Cross sectional area, Echo, Compression; ROC: Receiver Operating Characteristic curve; HRUS: High-resolution ultrasound; T2DM: Type 2 Diabetes; BMI: Body mass index; HbAlc: Glycosylated hemoglobin; V-R: Virchow-Robin space; UPSS: ultrasound pattern sum score; GBS: Guillain Barre syndrome.

## Introduction

Diabetic Peripheral Neuropathy (DPN) is one of the chronic complications of diabetes, mainly caused by sensory, motor and autonomic neurological dysfunction. At present 463 million adults worldwide are suffering from diabetes, and are expected to jump to 700 million by 2045. 4000–6000 patients have diabetic foot and lower extremity complications among them[1]. China is one of the prone areas of the disease. The latest data show that the prevalence rate of diabetes in China has risen to 11.2%. As a common complication, DPN incidence rate has increased year by year [2]. Due to the lack of doctors’ awareness of early diagnosis and people’s low awareness, most patients may have serious adverse consequences in the later stage, affecting the quality of life. In the early stage of DPN, small fiber neuropathy is often the main manifestation, which is characterized by symmetrical abnormal temperature and pain perception[3][4]. Among them, 25% of patients mainly seek medical treatment for the first time due to neuralgia[5], and some patients even die suddenly due to autonomic nerve dysfunction, which is an important indicator reflecting the prognosis of DPN patients; When the disease involves myelinated large fiber nerves, the patient may have numbness, weakened tendon reflex, abnormal walking and gait, or even distal limb muscle atrophyup to 50% of diabetic peripheral neuropathies may be asymptomatic[6], which brings great challenges to diagnosis and treatment. Therefore, it is very important to explore the early diagnosis of DPN and take effective treatment measures to improve the quality of life of patients. The pathogenesis is related to a variety of factors, which may be related to oxidative immune stress[7], accumulation of advanced glycokinase products[8, 9], metabolic disorders[10, 11], gastrointestinal flora disorders and immune factors[12]. At present, the exact etiology is not clear. 

Optimize glucose control and nutritional nerve are only to slow the progression of distal symmetric polyneuropathy in people with type 2 diabetes, but can not prevent the neurological impairment[6]. DPN is mainly diagnosed by clinical manifestations, electrophysiological testing or referral to a neurologistisrarely needed, except in situations where the clinical features are atypical or the diagnosis is unclear, due to lacking of sensitivity to small fibers[13]. With the development of ultrasound imaging, there are evidences that high-resolution ultrasound (HURS) plays a crucial role in the study of peripheral neuropathy. Many studies have confirmed that HURS has advantages in the diagnosis of early and subclinical lesions[14, 15], and makes up for the blank of morphological visualization. Nerves exposed permanently to high glucose and hypoxia environment are prone to axonal transport obstruction, resulted in insufficient nutrient supply of nerve endings[16].

Ultrasound imaging is still unspecific and difficult to distinguish the severity of the disease. Therefore, establishing the standard for evaluation of ultrasound is very critical for the early diagnosis of diabetic peripheral neuropathy. A few studies abroad have quantified the results of HRUS, such as Bochum ultrasonic score (BUS) [17], the ultrasonic pattern sum score (UPSS) [18], and study scoring system [19]. Although the diagnostic accuracy of HURS has been improved, this studies can not thoroughly quantify the ultrasonic evaluation. Therefore, the purpose of this paper was to comprehensively quantify the diabetic peripheral neuropathy by the “DCEC” scoring system and further confirm the diagnostic value.

## Materials and methods

### Study participants

This study was approved by the Affiliated Hospital of Guizhou Medical University in the Department of Neurology. Participants obtained written informed consents. None of the study results have been previously reported.

The study screened 289 consecutive patients with DPN, who were referred for the Chinese guidelines for the prevention and treatment of type 2 diabetes in 2017 between June 2016 and June 2020. Nerve conduction studies and ultrasonography were used for assessing neuropathies. Inclusion criteria was according to the 2017 Chinese guidelines for the prevention and treatment of type 2 diabetes, patients aged > 18 who meet the following criteria are included: fasting plasma glucose (FPG) in the morning ≥ 126 mg/dl (7.0mmol/l), 2-hour plasma glucose (2-h PG) ≥ 200 mg/dl (11.1mmol/l), or glycosylated hemoglobin ≥ 6.5% (48mmol/mol), or patients with symptoms of “excessive drinking, eating, urination and weight loss” + random peripheral blood glucose ≥ 200 mg/dl (11.1mmol/l)[13]. Patients were excluded if they had a history of peripheral neuropathy surgery, other infectious, immune, metabolic, poisoning, tumor, alcoholism, drugs and other causes of peripheral neuropathy; Patients with foot ulcer, severe cardiopulmonary insufficiency, obvious myasthenia, pregnancy, and so on[20, 21].

### Sample sizes

This study is a diagnostic retrospective study. The included patients are patients in the Department of Neurology. Therefore, most patients already have symptoms or signs, so it is difficult to obtain different grouping sample sizes according to the principle of sample size calculation. The sample size is grouped according to the actual situation of the included population.

**Fig. 1:**
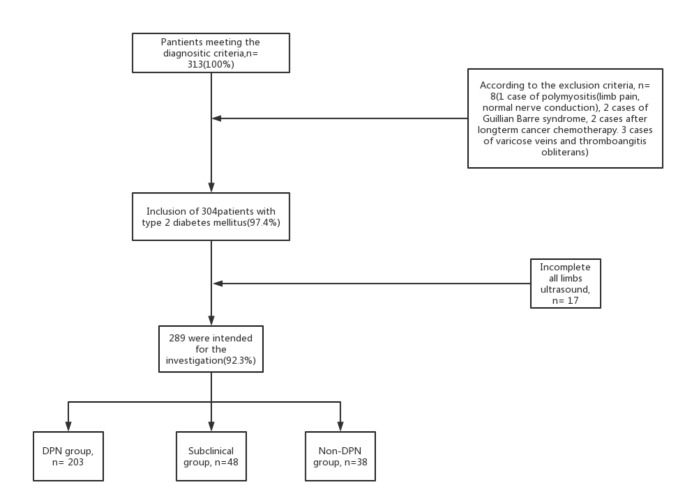
There were 313 patients with type 2 diabetes mellitus, including 1 case of polymyositis (limb pain, normal nerve conduction), 2 cases of Guillain Barre syndrome, 2 cases of long-term cancer, 3 cases of varicose veins and thromboangiitis obliterans after chemotherapy, and 16 cases without neuroelectrophysiological examination, so they were excluded. Finally, 289 patients with perfect limb neuroultrasound were included and divided into 3 groups, 20 cases in DPN group There were 3 cases, 48 cases in subclinical group and 38 cases in non DPN group

Grouping criteria: 203 patients with DPN symptoms or signs and abnormal neuroelectrophysiology were included in DPN group; The patients without symptoms and signs of peripheral neuropathy but with abnormal electrophysiological results were included in subclinical group (48 cases); 38 patients without symptoms and signs of peripheral neuropathy but normal nerve conduction were included in the non-DPN group[13](Fig. 1).

**Figure:**
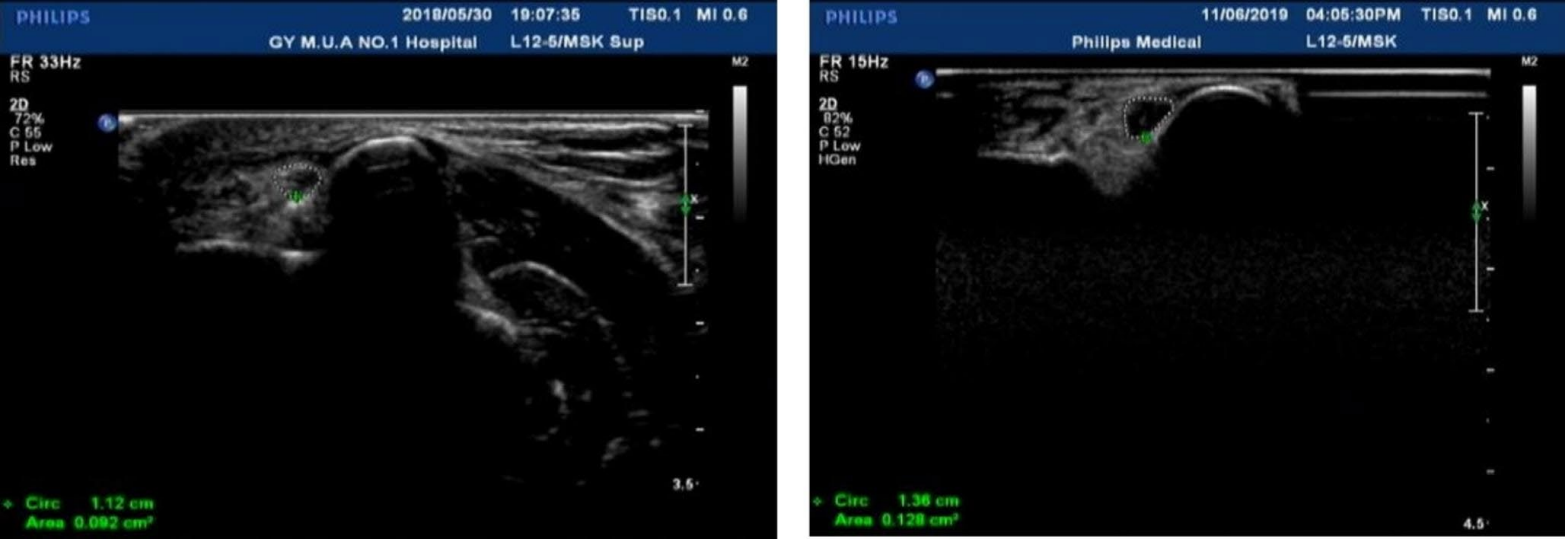
Cross sectional area of ulnar nerve in cubital tunnel under high frequency ultrasound.The cross-sectional area of normal ulnar nerve in cubital tunnel was 0.092cm^2^ (A); In the cross section of ulnar nerve in cubital tunnel of patients with diabetic peripheral neuropathy under high-frequency ultrasound, the internal nerve bundle structure was blurred, no hyperechoic nerve adventitia was found, and the cross-sectional area was 0. 128 cm^2^ (B)

### Diagnostic method

Some researches have confirmed the importance of ultrasound diagnosis in peripheral neuropathy[22–24]. Compared to neuro-electrophysiology, ultrasound not only has improved value and efficiency for diagnosing peripheral neuropathy, but helps distinguish the type of neuropathy based on morphological and structural changes[23]. Previously, we have reviewed that high-frequency ultrasound can diagnose diabetes peripheral neuropathy from the aspects of nerve cross-sectional area, blood flow, echo and compression. As the “gold standard” of Pathology, cutaneous nerve biopsy is difficult to popularize in clinical work due to the difficulty of obtaining materials and the high requirements of pathological diagnosis[24]. Studies have shown that the morphological changes of peripheral nerves in patients with type 2 diabetes occur before the occurrence of neuropathy, that indirectly reflected the diagnostic value of high frequency ultrasound[23, 25].Therefore, we chose the clinical diagnostic standard of diabetes peripheral neuropathy as the “gold standard” to measure the diagnostic value of high-frequency ultrasound score(DCEC).

### High-resolution ultrasound

Ultrasound was performed using a 5-12 MHz linear array transducer (Philips iU22, Holland) by a sonographer who was blinded to the groups of participants. The measurements should be perpendicular to the skin to avoid compressing nerves. The average values were taken for three times for each part. A total of 12 nerves in the limbs were detected by ultrasound. The cross-sectional areas(CSA) of each part of nerves should be measured along the hyperechoic boundary of nerves. Meanwhile, the definition and echo of nerves should be observed from multiple angles. The nerve entrapment, observed at wrist, elbow and ankle, should be recorded and described. Based on the range of normal nerve CSA in our laboratory, each site CSA value were abnormal if they exceeded the threshold value[14–18].

### Ultrasound scoring system

Referring to Ultrasonic scores of BUS, UPSS[19, 20], quantify the definition, cross-sectional area, echo, and compression of each nerve, and quantify the maximum value of this nerve measurement site. For detail, see the scoring system (Table 1).The Ultrasound scoring system (DCEC) is equal to total score (each score of nerve = definition score + cross-sectional score + echo score + compression score from each nerve), including the total score of both upper limbs and lower limbs; The above score was calculated by a neurologist.

**Table 1:** DCEC scoring system

Measurements	Neurological manifestations	Scoring
Definition	Clear	0
	Slightly fuzzy	1
	Vague	2
	Unclear	3
Cross-sectional area	normal	0
	≥ the normal value, ≤ 1.5 times of the normal value	1
	＞ 1.5 times of the normal value	2
Echo	Normal	0
	Increase or decrease	1
Compression	No	0
	Yes	1

Note: for each nerve, the range of clarity score [0,3], cross-sectional area score [0,2], echo score [0,1], compression score [0,1], total score range of each nerve [0,7], total score range of bilateral upper and lower limbs 12 nerves, total score range of limbs nerve [0,84]

### Nerve conduction studies

In a constant temperature environment, the Nicoli EDX EMG inducer were.

conducted in the electromyography laboratory at The affiliated Hospital of Guizhou Medical University in the Department of Neurology by an experienced technologist (Jing Huang). According to the diagnostic criteria of electrophysiological diagnosis of diabetic peripheral neuropathy[21], at least one abnormal NCS parameter in all nerves, we can figure out which limb has pathological changes.

### Statistical analysis

Statistical analyses were performed with SPSS software version 24. 0. The.

normal distribution of the data was tested with the Shapiro–Wilk test. Analysis of variance for continuous variables were used to compare variables with a normal distribution. Continuous variables were expressed as Interquartile Range due to not conform to normal distribution. Chi-square test and nonparametric Kruskal Wallis-H test was used for comparison among groups. A receiver operator characteristic curve (ROC) was generated by using the DCEC score datas (including measurements from the limbs), so that we can calculate the area under the curve (AUC) for DCEC total score, both upper limbs score, both lower limbs score, definition score,cross-sectional score, echo score, and compression score. Statistical significance was accepted at p < 0.05.

## Results

## Participants

313 patients with T2DM were enrolled in the study, but 24 patients were excluded, including 24 patients with incomplete datas, 16 cases without nerve conduction, and 1 patient with polymyositis, 2 patients with Guillain-Barre syndrome, 2 patients treated by cancer chemotherapy, 1 patients with polymyositis, and 3 patients with varicose veins and thromboangiitis obliterans. Thus, 289 were intended for the investigation, 153 (52.9%) male and 136 (47.1%) female, 93 (32.2%) had no symptoms of peripheral neuropathy but 196 (67.8%) had. In DPN group, 7 (3.4%) had no symptoms of peripheral neuropathy. It showed that age, gender, height, course of disease, BMI, fasting blood glucose, glycosylated hemoglobin level, smoking rate, hypertension, cerebral small vessel disease, dyslipidemia, overweight, poor blood glucose control, course of disease > 10 years and using of hypoglycemic drugs were without any statistical difference between groups(Table 2).

**Table 2:** baseline data of patients with type 2 diabetes mellitus

Clinical data	Type 2 diabetic patients (n = 289)	DPN group (n = 203)	Subclinical group (n = 48)	Non-DPN group (n = 38)	Inspection	P value
Age (years)	66 (57, 74)	66 (56, 74)	66 (55.8, 74.8)	66 (59.8, 72)	0.013	0.9938
Gender (male,%)	153 (52.9)	103 (50.7)	29 (60.4)	21 (55.2)	3.180	0.365
Height (cm)	161.3 (155, 168)	160 (155, 168)	165 (155, 169.8)	162.5 (154.8, 170)	3.413	0.182
BMI (kg/m^2^)	23.8 (21.8, 26)	23.9 (21.7, 26.0)	23.9 (21.9, 26.4)	23.3 (21.8, 25.1)	0.597	0.742
Course of disease (years)	7 (2, 10)	8.0 (2.0, 11.0)	6.0 (1.0, 11.0)	7.0 (2.75, 10.0)	1.802	0.406
Fasting blood glucose (mmol/L)	7.3 (6.1, 8.8)	7.5 (6.2, 9.2)	7.2 (6.0, 8.4)	7.1 (6.1, 8.7)	0.129	0.937
Glycosylated hemoglobin (%)	7.1 (6.5, 8.4)	7.5 (6.2, 9.2)	7.2 (6.5, 8.6)	7.0 (6.3, 7.7)	5.443	0.066
Smoking (yes,%)	109 (37.7)	77 (37.9)	18 (37.5)	14 (36.8)	0.017	0.991
The course of disease was ＞10 years	72 (24.9)	54 (26.6)	10 (20.8)	8 (21.0)	1.039	0.595
Medication (yes,%)	229 (79.2)	163 (80.2)	35 (72.9)	31 (81.5)	1.431	0.489
Metformin	99 (43.2)	69 (42.3)	13 (37.1)	17 (54.8)		
Insulin	79 (34.4)	57 (35.0)	13 (37.1)	9 (29.0)		
Metformin + insulin	28 (12.2)	20 (12.3)	6 (17.2)	2 (6.4)		
Other	23 (10.0)	17 (10.4)	3 (8.6)	9 (9.6)		

Abbreviations: BMI:Body Mass Index; DPN:Diabetic peripheral neuropathy

### Ultrasonographic features of peripheral neuropathy in patients with type 2 diabetes mellitus

Statistical comparisons among groups by one-way ANOVA. This difference in DCEC total score, clarity score, cross-sectional area score, radial nerve, ulnar nerve, sciatic nerve, tibial nerve, common peroneal nerve, total score of both upper limbs and total score of both lower limbs among groups was statistically significant (p < 0.05). The results revealed that there was no statistically significant difference between DPN group and Subclinical group after adjustment for multiple comparisons, but significantly higher than the non-DPN group (p < 0.05). The CSA score of subclinical group was higher than the DPN group (Table 3). However, echo and compression score were no significant difference among groups(Table 3). Then, we combined the DPN group and subclinical group into one group because no significant difference in multiple scores between the DPN group and subclinical group. Finally, results showed that the total score, definition score, CSA score, total score of upper extremity and total score of lower limbs from DCEC scoring system were higher than the non-DPN group. Among them, the definition score accounted for the highest proportion in the total score. Otherwise, the results of nerve and limb score were consistent with above among the groups. In DPN and subclinical group, the total scores of both lower limbs were higher than the total scores of both lower limbs. Among them, there were significant differences in ulnar nerve score, radial nerve score, sciatic nerve score, tibial nerve score and common peroneal nerve score among the groups (Table 4).

**Table 3:** DCEC score and its subgroup comparison

Group	DPN group (n = 203)	Subclinical group (n = 48)	Non-DPN group (n = 38)	Z value	P value
Definition score	11 (6, 17)^b^	11 (6.3, 16)^b^	4 (2, 8.3)	31.684	≤ 0.001
Cross sectional area score	4 (2, 7)^a^	5 (3, 8.8)^b^	3 (0.8, 5)	11.819	0.003
Echo scor	0 (0, 1)	0 (0, 1)	0 (0, 0.3)	0.171	0.918
Compression score	0 (0, 1)	0 (0, 1)	0 (0, 1)	0.644	0.725
Total score	17 (10, 24)^b^	18 (14, 22.8)^b^	8.5 (4, 16)	26.308	≤ 0.001

Note: Kruskal Wallis test, a showed that compared with subclinical group, the difference was statistically significant (P < 0.05); b showed that compared with the non DPN group, the difference was statistically significant (P < 0.05)

**Table 4:** Comparison of scores of nerves and limbs

Nerve score	DPN group (n = 203)	Subclinical group (n = 48)	Non-DPN group (n = 38)	Z value	P value
Median nerve score	4 (2, 5)	3 (2, 4.8)	2 (0, 4)	5.857	0.053
Ulnar nerve score	3 (1, 4)^b^	3 (2, 4)^b^	2 (0, 3.3)	8.705	0.013
Radial nerve score	1 (0, 3)^b^	1 (0, 2)^b^	0 (0, 1)	13.650	0.001
Sciatic nerve score	3 (1, 6)^b^	3 (2, 6)^b^	0.5 (0, 2.3)	24.832	≤ 0.001
Tibial nerve score	4 (2, 5)^b^	4 (2, 6)^b^	2 (0, 4)	18.426	≤ 0.001
Common peroneal nerve score	2 (0, 4)^b^	2 (0, 4)^b^	0 (0, 1.3)	17.872	≤ 0.001
Total score of upper limbs	8 (4, 12)^b^	8 (4, 10.8)^b^	4.5 (2, 8)	13.889	0.001
Total score of lower limbs	9 (5, 13)^b^	11 (6.3, 13)^b^	2 (1, 7.3)	29.221	≤ 0.001

Note: Kruskal Wallis test, a showed that compared with subclinical group, the difference was statistically significant (P < 0.05); b showed that compared with the non DPN group, the difference was statistically significant (P < 0.05)

### Diagnostic value of DCEC scoring system in DPN

**Fig. 2:**
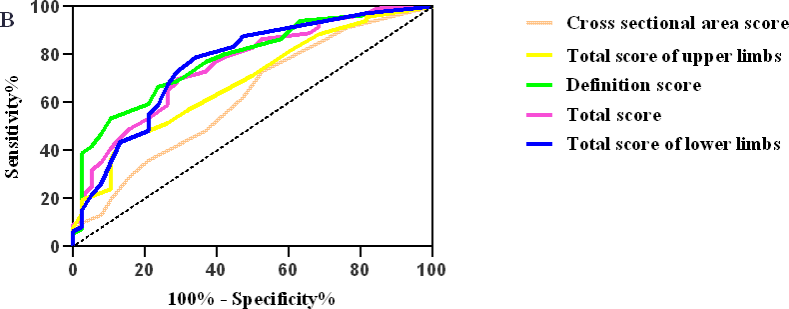
ROC curve of cross sectional area score, total score of upper limbs, difinition score, total score, and total score of lower limbs in diagnosis of DPN

Based on the diagnostic criteria of diabetic peripheral neuropathy as “gold standard”, the ROC curves from the ultrasound score according to Fig. 2. The cut-off values of DCEC total score, total score of both upper limbs, total score of both lower limbs and clarity score for DPN were 12.5 (sensitivity 69.7%, specificity71.1%). 8.5 (sensitivity 43.4%, specificity 86.8%), 4.5 (sensitivity 78.9%, specificity 65.8%), 10.5 (sensitivity 53.4%, specificity 89.5%), 2.5 (sensitivity 72.9%, specificity 47.4%) respectly. In addition, Their corresponding cross-sectional areas are 0.755(95% confidence intervals:67.5%, 83.6%), 0.687(95% confidence intervals:60.1%, 84.9%), 0.763(95% confidence intervals:67.7%, 84.9%), 0.783(95% confidence intervals:71.0%, 85.6%), 0.623(95% confidence intervals:52.6%, 72.0%)(Table 5).

**Table 5:** combined with DPN and subclinical group, the critical value of each score for diagnosis of DPN was obtained

DCEC score	The cut-off value (score)	sensitivity (%)	specificity (%)	The ratio of positive to negative	The ratio of negative to negative	Area under curve	P value
Total score	12.5	69.7	71.1	2.4	0.4	0.755 (0.675, 0.836)	≤ 0.001
Total score of upper limbs	8.5	43.4	86.8	3.3	0.2	0.687 (0.601, 0.773)	≤ 0.001
Total score of lower limbs	4.5	78.9	65.8	2.3	0.3	0.763 (0.677, 0.849)	≤ 0.001
Definition score	10.5	53.4	89.5	5.1	0.5	0.783 (0.710, 0.856)	≤ 0.001
Cross sectional area score	2.5	72.9	47.4	1.4	0.6	0.623 (0.526, 0.720)	0.015

Note: P < 0.05 indicates that the diagnostic cut-off value and ROC curve of the score are statistically significant

## Discussion

In order to improve the value of HRUS in the quantitative diagnosis of DPN, and avoid the error from “clinical electrophysiological separation” phenomenon of neurophysiology in the early stage of DPN[22], we diagnose DPN by clinical manifestations and neurophysiology, and analyzed DCEC scoring system in patients with T2DM. Finally, we found that there was no significant difference between DPN group and subclinical group. Compared with non-DPN group, DCEC score of DPN group / subclinical group was significantly higher. The results were consistent in several sensitivity analyses and subgroup analyses. We observed definition score is the most representative score in all group, and the cut-off value of DCEC score was 12.5, specially, the cut-off value of definition score was 10.5. This shows that we have made a new breakthrough in the diagnosis of diabetes peripheral neuropathy by high-frequency ultrasound, and improved the value of ultrasound diagnosis of diabetes peripheral neuropathy.

With respect to the ultrasound score between groups, definition scores were higher in all groups. The internal echo and definition of the nerves of the patients changed in varying degrees. The “cribriform or honeycomb” structure disappeared, with blurring development and boundary, and increase or decrease internal echoes. Difference in the scores between the subclinical group and the DPN group was insignificant, which indicates that the degree of nerve damage in type 2 diabetic patients may not be consistent with the clinical manifestations. Some patients may have peripheral neuropathy before the onset of diabetes or typical symptoms or signs. There was no significant difference between the neurological impairment in asymptomatic patients and patients with DPN. Even in some patients, the severity of peripheral neuropathy is similar to that of early diabetic neuropathy in prediabetes[23]. This may be determined by the severity of the neuropathy [24], which didn’t exclude that subclinical patients may even have more severe neuropathy.

In this study, the score of both lower limbs was higher than the both upper limbs, which was consistent with the “length dependence” of DPN, indicating that DPN patients had early involvement and severe damage of both lower limbs.

Several reports have shown that DCEC scoring system has high diagnostic value for peripheral neuropathy[25, 26]. The diagnostic value for diabetic peripheral neuropathy in this trial was as anticipated and was similar to that in previous studies. Using 12.5 points as the diagnostic cut-off value, the sensitivity and specificity of DPN were 69.7% and 71.1% respectively in this study, which isn’t higher than the diagnostic value presented by Qian OuYang et al[25]. But the definition score had a higher specificity (89.5%) in the diagnosis of DPN. We demonstrated that the definition score measured by HRUS could reflect the main nerve damage. The mechanism is that a large number of advanced glycation end products (ages) through protein kinase C (PKC) and transcription nuclear factor signaling pathway and other oxidative stress mechanisms in nerve tissue in hyperglycemia environment, causing excessive accumulation of sorbitol, increasing intracellular osmotic pressure, leading to edema of nerve tissue, demyelination or axonal degeneration [27, 28].

This study confirmed that the ultrasound score has certain advantages in reflecting the nerve damage in patients with diabetes peripheral neuropathy. Compared with the ultrasound pattern sum score, DCEC score focused more on the neuropathy quantification in limbs by HRUS, while the UPSS score focused on the quantification of motor, sensory and autonomic nerves[20]; In the measurement index, this study is not only limited to the nerve CSA, but also more comprehensive to evaluate the nerve definition, echo and compression.

Few studies paid attention to HRUS quantification, mainly in inflammatory immune related peripheral neuropathy recently. It suggested that CSA is a more consistent index for measurement as a quantitative index in most studies. In this study, we expanded the sample size to diagnose DPN in DPN, combined with nerve conduction to evade the “clinical electrophysiological separation” phenomenon of electrophysiology at the early stage of DPN[29]. It is more accurate and reliable to reflect the nerve lesions by HRUS.

The study has some limitations for the following reasons: (1) the proportion of patients without peripheral nerve symptoms is low because the study population mainly comes from the Department of Neurology, which caused the sample size is too small in subclinical and non-DPN group. There is no significant difference in the baseline data of patients, which can ensure the reliability of the results to a certain extent. Future long-term studies including larger cohorts of patients with type 2 diabetes mellitus from multiple departments and centers are needed, so that we can verify the reliability of DCEC score for DPN diagnosis.

### Conclusions

In conclusion, our results show that high frequency ultrasound can reflect the severity of peripheral neuropathy in diabetes and reliably diagnose DPN by DCEC score. Specially, the definition score is the most representative.

### Other information

The research project has been reviewed and approved by the ethics committee of the Affiliated Hospital of Guizhou Medical University, and registered with China Clinical Trial Registration Center (Registration No. chictr190021110).

### Data Availability

All data used to support the findings of this study are included within the article.

## Electronic supplementary material

Below is the link to the electronic supplementary material.

Supplementary Material 1

Supplementary Material 2
