# Supplementary material for: Sensitivity and specificity of high frequency ultrasound score (DCEC) in diabetic peripheral neuropathy
Source: J Diabetes Metab Disord. 2022 Aug 5;21(2):1459–67. doi: 10.1007/s40200-022-01080-6 (PMC9672188; doi:10.1007/s40200-022-01080-6)
Supplement: Supplementary file 1 — Supplementary Material 1 [file 40200_2022_1080_MOESM1_ESM.doc]

Reviewer #1

Question 1: Please explain why you evaluated the upper extremities and added the results to the final score. Was it necessary?

Answer 1: Thank you for your review. Multiple peripheral nerve damage is the most common type of peripheral neuropathy in diabetes, therefore, it is very important to clarify the peripheral nerve damage of the extremities. Measuring the nerves of both upper and lower limbs can more truly reflect the neuropathy of patients.

Question 2: Please estimate the cost and time required of neuro-ultrasound evaluation for each patient and mention it in the text ().

Answer 2: Thanks a lot for your question. The ultrasonic examination time of the peripheral nerves of each patient varies from 40 to 60 minutes according to the severity of the disease. It only needs high frequency ultrasonic instrument and couplant to complete, which has been supplemented in the red letter part from **High-resolution ultrasound** of the article.

Question 3: Please make clear the importance of this method in the practice. Is it a priority over previous reliable questionnaires? It seems that the importance of this study should be determined in comparison with other tools.

Answer 3: Many studies have confirmed the importance of ultrasound diagnosis in peripheral neuropathy. Compared to neuro-electrophysiology, ultrasound not only has improved value and efficiency for diagnosing peripheral neuropathy, but helps distinguish the type of neuropathy based on morphological and structural changes. Previously, we have reviewed that high-frequency ultrasound can diagnose diabetes peripheral neuropathy from the aspects of nerve cross-sectional area, blood flow, echo and compression. As the "gold standard" of Pathology, cutaneous nerve biopsy is difficult to popularize in clinical work due to the difficulty of obtaining materials and the high requirements of pathological diagnosis. Studies have shown that the morphological changes of peripheral nerves in patients with type 2 diabetes occur before the occurrence of neuropathy, that indirectly reflected the diagnostic value of high frequency ultrasound. Therefore, the current quantitative standard of ultrasonic diagnosis is particularly important.

Thank you for your suggestions. The answers to these questions are also explained in the red box.

Reviewer #2

Question 1: 1-Page 2, Line 35, Please indicate inclusion criteria clearly and list some of them.

Answer 1: Inclusion criteria was according to the 2017 Chinese guidelines for the prevention and treatment of type 2 diabetes, patients aged >18 who meet the following criteria are included: fasting plasma glucose (FPG) in the morning ≥ 126mg/dl (7.0mmol/l), 2-hour plasma glucose (2-h PG) ≥ 200mg/dl (11.1mmol/l), or glycosylated hemoglobin ≥ 6.5% (48mmol/mol), or patients with symptoms of "excessive drinking, eating, urination and weight loss" + random peripheral blood glucose ≥ 200mg/dl (11.1mmol/l). The above has been supplemented in the red letter part of the text.

Question 2: sample size calculation is missing, please add it as a sun heading and explain it in a paragraph.

Answer 2:

Sample size:

Actually, we have calculated the sample size before the study, but it’s extremely regrettable that the actual situation cannot be satisfied. This study is a diagnostic retrospective study. The included patients are patients in the Department of Neurology. Therefore, most patients already have symptoms or signs, so it is difficult to obtain different grouping sample sizes according to the principle of sample size calculation. The sample size is grouped according to the actual situation of the included population.

Question 3: Correct and arrange figure 2 (structure and words in the rectangles) and plot it in word in better resolution. also, see similar figures and complete it to the final step (outcomes, treatment).

Answer 3: Thank you for your suggestion. We have modified the resolution of Figure 2 (as shown in the figure). This study is a diagnostic study. Figure 2 shows the grouping process of this study, so there is no need for results and treatment process.

Question 4: plot figure 3 with a better size and beauty.

Answer 4: Thanks a lot to your advice. We have corrected that it is worthy to adjust the figure 3 with a better size and beauty.

Question 5: Check all the manuscript and use a space after . , : ; in incorrect cases.

Answer 5: Thank you very much for your suggestion. After careful checking, we have modified the punctuation in the text.

Question 6: check reference list and use a similar format based on the journal's guideline, for example any word in the title of the publication not to be capitalized:

Answer 6: Thank you for your reminder. We have revised the forms of references in the manuscript based on the journal's guideline.

Reviewer #3

Question 1: In order to bether report this article, I suggest to the authors to use the STARD 2015 guidelines for reporting diagnostic accuracy studies: explanation and elaboration.

Answer 1: Thank you for your suggestion. We have used the standard 2015 guidelines to report the diagnostic accuracy study to explain and elaborate this study.

Question 2: The Item 1. Identification as a study of diagnostic accuracy using at least one measure of accuracy (such as sensitivity, specificity, predictive values or AUC). Then I suggest to modify the title, it could be: Sensitivity and specificity of high frequency ultrasound score (DCEC) in diabetic peripheral neuropathy.

Answer 2: Your suggestion is very good. We have modified the title.

Question 3: Methods: How the sample size calculation was performed? Did the author´s perform a sample size calculation? Was this definition arbitrary? If so, give a rationaly about it.

Answer 3：We have explained the problem of sample size calculation in the red section of the manuscript. This study is a diagnostic retrospective study. The included patients are patients in the Department of Neurology. Therefore, most patients already have symptoms or signs, so it is difficult to obtain different grouping sample sizes according to the principle of sample size calculation. The sample size is grouped according to the actual situation of the included population. Actually, we have calculated the sample size before the study, but it’s extremely regretable that the actual situation cannot be satisfied.

Question 4: Statistical analysis: In the following statement: ''Continuous variables were expressed as the (25%, 75%) due to not conform to normal distribution'', I suggest to modify (25%, 75%) by Interquartile Range.

Answer 4: Thank you very much for your proposal. We have amended it in the red section of the document.

Question 5: Results: The authors need to present the estimates of diagnostic accuracy and their precision (such as 95% confidence intervals).

Answer 5: Thank you for your suggestions. The estimation of diagnostic accuracy and its accuracy have been supplemented in the text (see the red letter part). In addition, Their corresponding cross-sectional areas are 0.755(95% confidence intervals:67.5%, 83.6%), 0.687(95% confidence intervals:60.1%, 84.9%), 0.763(95% confidence intervals:67.7%, 84.9%), 0.783(95% confidence intervals:71.0%, 85.6%), 0.623(95% confidence intervals:52.6%, 72.0%)(Table 5).

Question 6: Conclusion: The following statement is a little confusing: In conclusion, our results showed that high frequency ultrasound can energize the peripheral neuropathy of diabetic peripheral neuropathy. So, I suggest to the author to review and rewrite this sentence.

Answer 6: Thanks a lot for your reminder. We have modified it in the manuscript and marked it in red.

Answer #4:

Question 1: Abstract: - Please state the study design in the methods.

Answer 1: The study design methodology has been supplemented in the summary methodology.

Question 2:

Introduction

- The pathogenesis is still uncertain recently (add the reference).

- The main idea in the introduction is not structure. Please separate into some paragraphs.

- What is the urgency of DPN? please state clearly.

- can you briefly state the novelty of the study?

- why the study focuses on Ultrasound imaging? Meanwhile there are many diagnostic treatment to assess the DPN. Please state it.

Answer 2: The currency of DPN has been stated clearly at the red mark of the currency of DPN in the introduction.

Diabetic Peripheral Neuropathy (DPN) is one of the chronic complications of diabetes, mainly caused by sensory, motor and autonomic neurological dysfunction. At present 463 million adults worldwide are suffering from diabetes, and are expected to jump to 700 million by 2045. 4000-6000 patients have diabetic foot and lower extremity complications among them[1]. China is one of the prone areas of the disease. The latest data show that the prevalence rate of diabetes in China has risen to 11.2%. As a common complication, DPN incidence rate has increased year by year. Due to the lack of doctors' awareness of early diagnosis and people's low awareness, most patients may have serious adverse consequences in the later stage, affecting the quality of life. In the early stage of DPN, small fiber neuropathy is often the main manifestation, which is characterized by symmetrical abnormal temperature and pain perception. Among them, 25% of patients mainly seek medical treatment for the first time due to neuralgia, and some patients even die suddenly due to autonomic nerve dysfunction, which is an important indicator reflecting the prognosis of DPN patients; When the disease involves myelinated large fiber nerves, the patient may have numbness, weakened tendon reflex, abnormal walking and gait, or even distal limb muscle atrophyup to 50% of diabetic peripheral neuropathies may be asymptomatic[3], which brings great challenges to diagnosis and treatment. Therefore, it is very important to explore the early diagnosis of DPN and take effective treatment measures to improve the quality of life of patients.

This study is the first time to quantify the damage of diabetes peripheral neuropathy by high-frequency ultrasound, which is a new leap in the study of diabetes peripheral neuropathy by high-frequency ultrasound.

Question 3:

Methods

- The study design is not well stated.

- Do you used formula to obtain the total sample?

- The inclusion criteria is not clear. ADA criteria may be not specific for readers and needs effort to find on ADA criteria.

- Do you have any age classification in this study? adult? older adult? that recruited in the study

- Why PN surgery is excluded? Poisoning? What type of poison? location?

- Regarding the intervention using High-resolution ultrasound, how do you manage adverse or sentinel event?

- It would be full of information, if you present in figure the spot of nerves while assessing using HRU.

- Please present the calculation, classification, and how to total the scoring of ultrasound scoring system.

- "In a constant temperature environment" how many degree Celsius?

- Please state the study protocol in the new sub-heading.

Answer 3: The diagnosis of diabetes peripheral neuropathy is mainly based on the clinical symptoms and signs of the patients, and the neuroelectrophysiological examination is not a necessary condition. Due to the lack of sensitivity to small fiber nerves, the missed diagnosis rate of early diagnosis is high. The American ADA guidelines in 2020 recommend that it should be improved only for those who are atypical or difficult to diagnose. Skin biopsy can effectively diagnose peripheral neuropathy through the damage of epidermal nerve fibers. It is difficult to popularize in clinical work due to the difficulties in obtaining materials and the high requirements for pathological diagnosis. In recent years, with the development of ultrasound imaging technology, high-frequency ultrasound plays an increasingly important role in the diagnosis of peripheral neuropathy. Because of its high resolution, ultrasound can clearly show the hierarchical structure of the inner, fascicular and outer membranes of nerve, which has been proved by many studies to have unique advantages in the diagnosis of early and subclinical peripheral neuropathy. In the diagnosis of diabetes peripheral neuropathy, it makes up for the blank of morphological visualization in the study of neuroelectrophysiology, and improves the early diagnostic value of patients.

Actually, we have calculated the sample size before the study, but it’s extremely regrettable that the actual situation cannot be satisfied. This study is a diagnostic retrospective study. The included patients are patients in the Department of Neurology. Therefore, most patients already have symptoms or signs, so it is difficult to obtain different grouping sample sizes according to the principle of sample size calculation. The sample size is grouped according to the actual situation of the included population.

Thank you for your suggestions. We believe that the ADA guidelines can not accurately describe the diagnostic criteria of diabetes. According to the specific situation of China, the diagnostic criteria of type 2 diabetes in the 2017 guidelines for the prevention and treatment of type 2 diabetes are adopted.As follows:

Inclusion criteria was according to the 2017 Chinese guidelines for the prevention and treatment of type 2 diabetes, patients aged >18 who meet the following criteria are included: fasting plasma glucose (FPG) in the morning ≥ 126mg/dl (7.0mmol/l), 2-hour plasma glucose (2-h PG) ≥ 200mg/dl (11.1mmol/l), or glycosylated hemoglobin ≥ 6.5% (48mmol/mol), or patients with symptoms of "excessive drinking, eating, urination and weight loss" + random peripheral blood glucose ≥ 200mg/dl (11.1mmol/l).

 All the people included in the study are adults over 18 years old. All the people included in this study are over 50 years old and all of them are patients in our department.

 On the one hand, peripheral neuropathy is prone to adhesion of bony ducts or fibrous connective tissue, which affects our judgment of neuropathy itself; In addition, according to the guidelines, peripheral neuropathy caused by other causes should be excluded in the diagnosis of diabetes peripheral neuropathy. Among them, toxic contact can cause damage to the peripheral nerve axon, such as some anti-tumor drugs and antiviral drugs. Therefore, it should also be excluded.

As an examination tool for peripheral neuropathy, high-frequency ultrasound has the characteristics of non-invasive, simple and easy to operate, and visual lesions. Our team has 10+ years of clinical practice experience, and is in a leading position in China. No adverse events have been found. No adverse events or sentinel events have been found by consulting relevant literature.

Referring to Ultrasonic scores of BUS, UPSS[19, 20], quantify the definition, cross-sectional area, echo, and compression of each nerve, and quantify the maximum value of this nerve measurement site. For detail, see the scoring system (Table 1).The Ultrasound scoring system (DCEC) is equal to total score (each score of nerve= definition score + cross-sectional score + echo score + compression score from each nerve), including the total score of both upper limbs and lower limbs; The above score was calculated by a neurologist.

**Table. 1 DCEC scoring system**

| Measurements | Neurological manifestations | Scoring |
| --- | --- | --- |
| Definition | Clear | 0 |
|  | Slightly fuzzy | 1 |
|  | Vague | 2 |
|  | Unclear | 3 |
| Cross-sectional area | normal | 0 |
|  | ≥ the normal value, ≤ 1.5 times of the normal value | 1 |
|  | ＞ 1.5 times of the normal value | 2 |
| Echo | Normal | 0 |
|  | Increase or decrease | 1 |
| Compression | No | 0 |
|  | Yes | 1 |

Note: for each nerve, the range of clarity score [0,3], cross-sectional area score [0,2], echo score [0,1], compression score [0,1], total score range of each nerve [0,7], total score range of bilateral upper and lower limbs 12 nerves, total score range of limbs nerve [0,84].

Refer to the book rehabilitation assessment，the temperature will affect the nerve conduction velocity, thus, the room temperature should be carried out in a constant temperature environment (34 ℃), when conducting nerve conduction examination.

Question 4:

Results

- overall is clear, but, please present one-way anova analysis in the "statistical analysis" methods.

 Answer 4: Thanks a lot for your advice on statistics. In this study, there are more than 3 groups, and each data does not conform to the normal distribution. However, the one-way ANOVA is mainly used for the t-test of two independent samples that conform to the continuous normal distribution. Therefore, this method is not suitable for this study.

Question 5:

Discussion

- overall is clear. Please state the strength, limitation and implication practice.

- the value of percentage should not be presented in the discussion. Please delete and present the meaning of value in the discussion.

- Please add further suggestions.

Answer 5:

Strength

This study confirmed that the ultrasound score has certain advantages in reflecting the nerve damage in patients with diabetes peripheral neuropathy. Compared with the ultrasound pattern sum score, DCEC score focused more on the neuropathy quantification in limbs by HRUS, while the UPSS score focused on the quantification of motor, sensory and autonomic nerves[20]; In the measurement index, this study is not only limited to the nerve CSA, but also more comprehensive to evaluate the nerve definition, echo and compression.

Limitation

The study has some limitations for the following reasons: (1) the proportion of patients without peripheral nerve symptoms is low because the study population mainly comes from the Department of Neurology, which caused the sample size is too small in subclinical and non-DPN group. There is no significant difference in the baseline data of patients, which can ensure the reliability of the results to a certain extent. Future long-term studies including larger cohorts of patients with type 2 diabetes mellitus from multiple departments and centers are needed, so that we can verify the reliability of DCEC score for DPN diagnosis.

Implication

This shows that we have made a new breakthrough in the diagnosis of diabetes peripheral neuropathy by high-frequency ultrasound, and improved the value of ultrasound diagnosis of diabetes peripheral neuropathy.

The value of this study has been stated in the red letter of the first paragraph of the discussion section.
