# Supplementary material for: Sensitivity and specificity of high frequency ultrasound score (DCEC) in diabetic peripheral neuropathy
Source: J Diabetes Metab Disord. 2022 Aug 5;21(2):1459–67. doi: 10.1007/s40200-022-01080-6 (PMC9672188; doi:10.1007/s40200-022-01080-6)
Supplement: Supplementary file 2 — Supplementary Material 2 [file 40200_2022_1080_MOESM2_ESM.doc]

**Figure Legend**

**Figure 1 Cross sectional area of ulnar nerve in cubital tunnel under high frequency ultrasound.The cross-sectional area of normal ulnar nerve in cubital tunnel was 0.092cm^2^ (A); In the cross section of ulnar nerve in cubital tunnel of patients with diabetic peripheral neuropathy under high-frequency ultrasound, the internal nerve bundle structure was blurred, no hyperechoic nerve adventitia was found, and the cross-sectional area was 0. 128 cm^2^ (B).**

**Figure 2 There were 313 patients with type 2 diabetes mellitus, including 1 case of polymyositis (limb pain, normal nerve conduction), 2 cases of Guillain Barre syndrome, 2 cases of long-term cancer, 3 cases of varicose veins and thromboangiitis obliterans after chemotherapy, and 16 cases without neuroelectrophysiological examination, so they were excluded. Finally, 289 patients with perfect limb neuroultrasound were included and divided into 3 groups, 20 cases in DPN group There were 3 cases, 48 cases in subclinical group and 38 cases in non DPN group.**

**Figure. 3 ROC curve of cross sectional area score, total score of upper limbs, difinition score, total score, and total score of lower limbs in diagnosis of DPN.**
